# The Effectiveness of Blue-Light-Emitting Glasses in Security Guards Exposed to Night Shift Work on Work-Related and General Fatigue: A Randomised Controlled Cross-Over Study

**DOI:** 10.3390/clockssleep4040051

**Published:** 2022-11-24

**Authors:** Pieter H. Helmhout, Stella Timmerman, Alwin van Drongelen, Eric W. P. Bakker

**Affiliations:** 1 Training Medicine and Training Physiology, Directory of Personnel, Army Command, Royal Netherlands Army, P.O. Box 90004, 3509 AA Utrecht, The Netherlands; 2 Radboudumc Health Academy, Radboud University, P.O. Box 9101, 6500 HB Nijmegen, The Netherlands; 3 Safety and Human Performance, Royal NLR–Netherlands Aerospace Centre, P.O. Box 90502, 1006 BM Amsterdam, The Netherlands; 4 Department of Epidemiology and Data Science, Division EPM, University Medical Center Amsterdam, P.O. Box 22600, 1100 DD Amsterdam, The Netherlands

**Keywords:** blue light, shift work, need for recovery, randomized controlled trial, field-based study

## Abstract

This study aimed to evaluate the effectiveness of glasses that emit blue light in reducing the need for recovery, general fatigue, and stress levels in security guards who work night shifts. Light manipulation is seen as a promising strategy to mitigate complaints related to shift work, such as sleepiness and impaired cognitive performance. In a randomized controlled cross-over study design, 86 Dutch security guards used light-emitting glasses (exposure duration: 30 min) during night shifts in a five week period versus a five week control period without glasses. Measurements (Need for Recovery Scale; Checklist Individual Strength; stress level assessed by a fitness tracker) were performed at baseline, at five weeks, and again at 11 weeks. The chronotype was measured at baseline as a potential covariate. A mixed model for repeated measure analyses showed no significant reduction in the need for recovery, nor a reduction in general fatigue scores, during the intervention period. Paired Samples T-Test analyses showed no significant changes in stress levels for the intervention period. Conclusively, blue light exposure using light-emitting glasses for security guards during night shifts showed no directly measurable effect on the reduced need for recovery, overall fatigue, and stress levels.

## 1. Introduction

Shift workers form a substantial part of the Western working population. Approximately one-fifth of the total labour force in the European Union are exposed to working shifts [1]. In the Netherlands, around 14% of the currently active Dutch population regularly works in shifts, of which the large majority is often exposed to night work [2].

Shift work, including night work, is known to dysregulate the circadian clock [3], the body’s internal system that is responsible for day and night rhythms of crucial biological processes such as body temperature, hormone regulation, and the sleep-wake cycle [4]. The circadian clock needs time to adjust to being active at night, but this time is typically lacking when working in rotating shifts. This renders shift workers 1.5 to 2 times more prone to developing fatigue, sleepiness, and sleeping problems, both at daytime and at night, compared to people who have a regular day job [5,6].

Consequently, work-related accidents and injuries are significantly higher in shift workers compared to workers in regular day jobs [7,8]. Night shift exposure compromises both short-term and long-term physical, social, and cognitive performance, and can include impairments in visual motor skills, increased irritability, declined vigilance, and short-term memory loss [8,9,10]. These effects persist on the long term, and become more pronounced in daily life after ten or more years of exposure to night shifts [11]. Moreover, evidence is building that long-term shift work is related to chronic health problems such as respiratory infections, diabetes mellitus (type 2), cardiovascular disease, and cancer [12,13].

The Dutch Society of Occupational Medicine has recently published guidelines on promising strategies to mitigate the negative consequences of shift work [14]. These guidelines include good sleep hygiene, adjusting work schedules, power naps, nutritional adjustments, and light manipulation. The levels of evidence for these different strategies differ: the guidelines underline the benefits of good sleep hygiene and napping, whereas the evidence of light manipulation is small and inconclusive, possibly due to the large heterogeneity of the studies involved with respect to the different modes of light exposure used, different timing strategies, et cetera. As a result of this lack of evidence, the current guidelines do not provide advice on whether or how to incorporate light manipulation into shift work strategies.

The feasibility of light-based interventions over often more complex institutional changes, such as modifying work schedules or allowing night time naps, has been emphasized [15]. Light manipulation is seen as a potentially powerful non-pharmacological strategy to manipulate melatonin production and, thus, better align circadian rhythms with shift work schedules [16]. Nevertheless, the usefulness of shifting circadian rhythm in rapidly rotating shift workers has been questioned [17], and the health effects of long-term adjustment of the circadian rhythm through light-induced melatonin suppression remain largely unknown.

In addition to a phase effect, the alerting effect of low-intensity light manipulation may acutely and temporarily mitigate complaints related to shift work, such as sleepiness and impaired cognitive performance [17,18]. Light manipulation has been studied in different shift work settings, such as hospitals [15,18,19,20,21,22], police cars [23], and operator control rooms [24]. Most studies involve either environmental light adjustments or the usage of light boxes. Two recent studies have focused on the effectiveness of light manipulation in hospital nurses using personal light-emitting glasses with specific light frequencies [19,20].

Compared to environmental bright light and light boxes, the light-emitting glasses that are commercially available nowadays may have some additional potential benefits regarding ease of use, personalization, and exposure stability irrespective of head position [25]. Positive effects of light exposure via light-emitting glasses have been found in athletes [26]; in patients with sleep disorders [27], depression [28], and Parkinson’s disease [29]; and in survivors of severe traumatic brain injury [30]. The few studies conducted thus far that have addressed shift work populations show inconclusive results concerning the effectiveness and applicability of light-emitting glasses [19,20].

To our knowledge, no controlled field-based study has been performed on the use of light-emitting glasses in shift work security service settings. Security settings demand a continuous level of vigilance combined with, depending on job function and location, low cognitive and physical stimuli, a combination that is prone to induce sleepiness at night time. A recent unpublished inventory amongst civilian security guards from the Netherlands Armed Forces Surveillance and Security Organization showed that issues concerning work stress, fatigue, and the need for recovery from work are a burden to this group of employees, due to the combined impact of rapidly rotating (night) shift work and shortage of personnel.

Recent research suggests beneficial effects of light-based interventions for reducing fatigue in rapidly rotating shift workers over time [15]. Therefore, the aim of this study was to evaluate the effectiveness of commercially available light-emitting glasses for security guards exposed to night shift work. It was hypothesized that the alertness effect of low-intensity light exposure during the shift would have a positive effect on the individual’s need for recovery and both general and work-related fatigue.

## 2. Results

### 2.1. Participants and Missing Data

Eighty-seven service guards met the inclusion criteria and were included in the study. The mean age (standard deviation, SD) of the predominantly male (94%) baseline group was 39.6 (8.1) and ranged between 21 and 60 years. The mean number of years of working in a security service setting was 15.3 (8.7), ranging between 1 and 38 years. Three participants (3%) reported they had been using melatonin as a sleep aid in the last month before study entry; seven subjects (8%) used additional sleep aids (e.g., a sleep apnoea device, cannabidiol oil), and nine participants used sleep medication prescribed by their general practitioner. Detailed participant characteristics are presented in Table 1. Group 1 (G1) started with a five week intervention period using light-emitting glasses during night shifts, followed by a five week control period without glasses; group 2 (G2) started with the control period, followed by the intervention period.

All participants completed each measurement at T_0_, T_5_, and T_11_, with the exception of one, who dropped out before the last measurement in week 11 due to absenteeism unrelated to the study.

In total, 14 participants (16%) answered 25 questionnaire items incorrectly: 14 for the Need For Recovery Scale (NFRS) and 11 for the Checklist Individual Strength (CIS). All missing questionnaire data were considered missing not at random (MNAR). For the NFRS, missing data could likely be ascribed to the dichotomous character of the scale, forcing participants to make a choice between two badly fitting options. For the CIS, missing data were seemingly related to the positioning of specific questions in the printed version of the questionnaire.

Due to technical problems with downloading the stress data from the fitness trackers onto the data platform, we missed data from all participants. In total, 42% of the stress data at T_5_ and 7% of the stress data at T_11_ went missing completely at random (MCAR), as there was no way to trace back if stress data were lost before or during the downloading process. The average stress levels were calculated when a minimum of 480 data points were available, representing two days of data. Using this threshold, 64 participants (74%) were included in the statistical analysis.

### 2.2. Outcomes

The mean scores and standard deviations of the NFRS, CIS, and stress level, for both groups at the three time points, are presented in Table 2 and Table 3.

**Figure 1:**
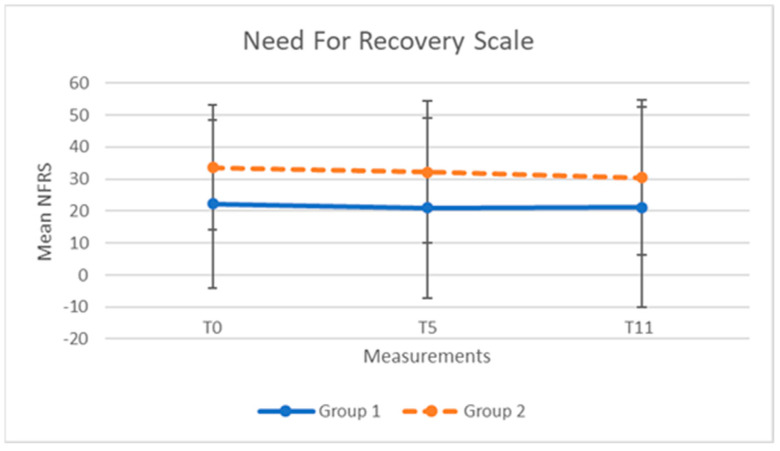
NFR scores (mean, SD) for group 1 (N = 45) and group 2 (N = 42) at baseline (T_0_), after 5 weeks (T_5_), and after 11 weeks (T_11_).

**Figure 2:**
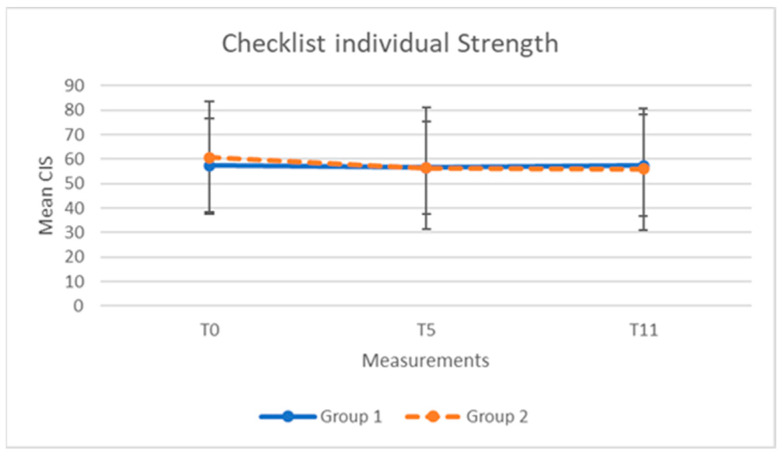
CIS scores (mean, SD) for group 1 (N = 45) and group 2 (N = 42) at baseline (T_0_), after 5 weeks (T_5_), and after 11 weeks (T_11_).

**Figure 3:**
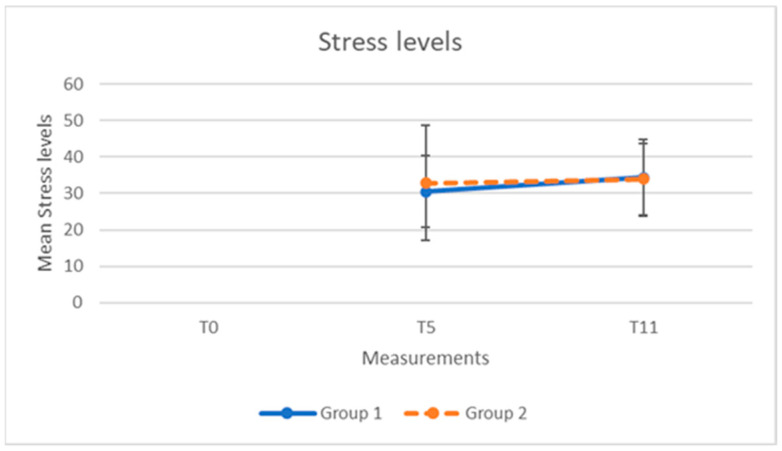
Stress levels (mean, SD) for group 1 (N = 45) and group 2 (N = 42) at 5 weeks (T_5_) and at 11 weeks (T_11_).

For both groups, the scores of NFRS, CIS, and stress over time are shown in Figure 1, Figure 2 and Figure 3.

The results for the group-related and time-related intervention effects in the NFRS and CIS revealed no significant interaction effects (Table 4). Neither the main effects of age and years in service, nor their interactions with time or intervention sequence, were significant. No indication of an order effect on the effect of the light glasses was found: differences between mean scores after five weeks of using light glasses or after a five week control period were not significant for both NFRS (−0.2; *p* = 0.54) and CIS (−0.5; *p*= 0.96). The Akaike information criterion (AIC) values for goodness of fit of the mixed-effects model with random intercept were 2217.7 for the NFRS and 2055.8 for the CIS.

The average (SD) scores after five weeks of wearing glasses were 25.5 (28.0) for the NFRS and 56.2 (22.6) for the CIS. The scores after the five week control period were 26.6 (25.8) for the NFRS and 57.1 (22.0) for the CIS. The Estimated Cohen’s effect sizes were not significant for both the NFRS (0.08; 95% CI: −013 to 0.29) and the CIS (0.09; 95% CI: −0.12 to 0.30).

The results for stress level showed that the use of light-emitting glasses during a night shift did not significantly reduce stress level values (mean 1.45; SD 11.47; *p* value 0.315) in the study group.

### 2.3. Adherence to Intervention Protocol

Thirty participants (35%) wore the light-emitting glasses during seven or more night shifts; 11 participants (13%) during six shifts; 17 participants (20%) during five shifts; and 28 participants (33%) during four or less shifts. Changes within the shift work schedules of some participants have led to deviations from the number of night shifts during a standard five week block period, with eight or nine shifts in seven cases, instead of the protocolled seven shifts. The glasses were used, on average, at 5.3 night shifts (SD = 1.8; range 1–9). Overall, the light-emitting glasses were used 257 (43%) out of 602 night shifts. Several reasons for lack of adherence to the intervention protocol were given: ad hoc changes to the work schedule, leave days, sick leave, and missing app notifications due to technical issues.

### 2.4. Experiences of the Study Participants

After the study, 31 participants (36%) were motivated to use the light-emitting glasses more often on duty, while 17 participants (20%) indicated they needed to test the glasses for a longer period before they were able to make a final judgment on its potential benefits. 38 participants (44%) reported that they had no intention of using the light-emitting glasses in the future, mainly due to a lack of noticeable positive effects. Seven participants reported some adverse events when using the glasses (e.g., dry eyes and/or light headache), which caused one participant to decide to stop wearing the glasses during the remaining shifts. For the other six subjects, removing the blue-tinted lenses when wearing the glasses in on-modus gave enough relief to continue wearing them during the rest of the intervention period. A rare, but relevant, side effect reported by two participants was the more pronounced tendency to nod off during the ride home when using the glasses at the preceding night shift. In contrast, three participants reported to have, occasionally, more trouble falling asleep after a shift when they had used the light-emitting glasses.

## 3. Discussion

The aim of our study was to evaluate the effectiveness of commercially available blue-light-emitting glasses, in terms of recovery need, fatigue, and related behavioural aspects, in security guards exposed to night shift work. Lower health and wellbeing levels have been reported in night security guards, including higher occurrences of occupational stress, sleep disturbances, and work-related fatigue [31,32,33]. A recent systematic review underlined the potential of bright light exposure in shift workers, but emphasized the need for more and larger randomized controlled trials [34]. We therefore investigated whether blue light exposure during the night shift could decrease work-related and general fatigue and stress levels in a group of 86 security guards. The overall results of our study did not show significant beneficial effects of nocturnal light exposure in our study group. The effect sizes of the study intervention were small and not significant.

A recent study among hospital nurses by Van Woerkom [19], using similar light exposure duration per shift, also found no positive effects of light-emitting glasses on fatigue and psychological well-being after three night shifts. A positive effect was only found when light-emitting glasses and napping were combined. Another recent study using light-emitting glasses among hospital nurses did show some positive effects on different scales for sleepiness, most prominently in the first of three consecutive night shifts [20]. Light exposure was administered four times, for 15 min, at midnight, 1 am, 2 am, and 3 am, respectively, and for 30 min within 2 h after waking up. Thus, light exposure in this study was significantly longer, suggesting that exposure duration may influence the effectiveness of the light intervention. Moreover, light exposure may have had more prominent and measurable effects on the momentary sleepiness and vigilance parameters that were used in this study compared to outcomes related to longer term fatigue and well-being, as in our study.

Others have suggested the importance of exposure duration. Two studies with a long exposure duration (10 to 12 consecutive hours) found positive effects of bright environmental light in reducing sleepiness among intensive care nurses and control room workers during a 12 h night shift [21,24]. However, another long-exposure study (five consecutive hours) in police patrol officers found no effects on sleepiness [35], while several recent studies in nurse populations, with relatively short exposure times comparable to our study (40 min or less), showed mixed results: Aarts et al. [20] and Olsen et al. [16] reported positive effects on sleepiness, while no effects were found in studies by Bjorvatn et al. [18] and Van Woerkom [19]. Definitive conclusions on optimal exposure duration can therefore not be drawn, also because of the different light intensity levels that were used in these studies.

Light-emitting glasses have a lower light intensity compared to light boxes or environmental light, but they are positioned closer to the eyes. A laboratory study tested both glasses (2000 lux) and boxes (10,000 lux) and found similar positive effect for changes in sleepiness and vigilance parameters [27]. However, light-emitting glasses had a greater prolonged effect on vigilance after 2 h of enriched bright light exposure compared to light boxes. The glasses used in our study produced 35 lux, which was similar to those used in other studies [19,20]. Still, findings were contradicting, suggesting that exposure intensity alone is not determinative for the effectiveness of nocturnal light exposure. As recently suggested, melanopic equivalent daylight illuminance (EDI) may be a more valid determinant of melatonin suppression effects in light glasses [36,37]. Aarts et al. [20] reported an EDI level of 114 lux provided by the light glasses used in our study, which should induce phase-shifting and alerting effects after 1 h. This suggests that the light exposure protocol of our study, as provided by the manufacturer, may have been too low to be effective.

Some methodological aspects concerning the selection of outcomes and study group characteristics may have contributed to the lack of results found in the current study. Our primary outcome measure, NFRS, represents self-assessed fatigue and need for recovery in the preceding 14 days. As mentioned, outcomes related to longer term fatigue and well-being may be less sensitive to change than momentary sleepiness and vigilance parameters measured during, or shortly after, the night shift. From an organizational point of view, lasting improved levels of fatigue and the need for recovery among employees can be more relevant than acute and short-term effects on sleepiness, as they are more strongly related to job satisfaction, work absenteeism, or intention to leave the job [35].

Regarding study group characteristics, floor effects may have led to the lack of findings in our study. First, NFRS has been shown to be age-dependent: aging shift workers (≥45 years) show a significant higher need for recovery compared to younger shift workers (<45 years) [38,39]. In our study population, age ranged between 21 and 60 years, but with the majority of participants (74%) being younger than 45. The sensitivity of our primary outcome may, thus, have been too low to detect significant intervention effects in this study group.

Second, with a mean NFRS score of 27.6 (23.7), our study group showed lower levels of the need for recovery than those reported in reference populations. In the Maastricht Cohort Study [34], men and women showed average NFRS scores of 45.7 and 42.8, respectively, both controlled for age and long-term disease. In another study [40], a group of nurses who experienced sleep-related complains had a mean NFRS ranging between 60 and 62, while those without sleep-related complaints still scored significantly higher (between 45 and 49) compared to our baseline NFRS scores. We included in our field-based study all guards that met the inclusion criteria and were motivated to experience the light-emitting glasses during their night shifts, without assessing whether they had issues related to shift work such as fatigue or sleep disturbances. Choosing a selected group of guards with actual problems in this respect, i.e., with potentially higher NFRS scores, might have revealed more clear effects.

Third, it seems unlikely that gender plays a crucial role in (blue) light exposure during night shifts, as both our predominantly male study group and female-dominant nurse populations [17,19] show inconsistent results regarding the effectiveness of the intervention. The relatively high level of work experience in our study group (15 years on average), on the other hand, may have influenced the effectiveness of the intervention under study. Following the healthy worker effect, more experienced shift workers may have developed better coping strategies to deal with the negative consequences of their work than shift workers that quit earlier in their career, thus being more ‘immune’ to new interventions such as nocturnal light exposure [17]. However, years of experience was not found to be a significant covariate in our analyses (data not presented).

The design of our study has some strengths and limitations. We used a relatively robust, though somewhat underpowered, controlled study design in a group of actual shift-working individuals. The randomization process was intended to ensure that expected variations in potential influencing factors, such as individual coping strategies (e.g., caffeine/food intake, sleep strategies prior to/following night shifts), were evenly distributed between the two intervention groups. To maintain conditions close to daily practice, we emphasized in our pre-study instructions that participants were allowed to continue using their personal strategies during the study, both at work and during time off work.

This field-based approach may have introduced several limitations. Neither the daytime light exposure preceding the night shift nor the lighting environment at the security locations were regulated or monitored. Earlier research indicates that light exposure history can make shift workers more insensitive to additional nocturnal light exposure. One field-based study showed that nocturnal light exposure was more effective in suppressing melatonin levels in a group previously exposed to a week of dim light, compared to a group that was exposed to a week of bright light [41]. A lab-controlled study with participants who were awake for 24 h showed positive effects of nocturnal light exposure, via glasses and boxes, compared to ambient dim light, on reduced sleepiness and increased vigilance [27]. All locations in our study used ambient dim lighting at night.

Furthermore, our study was performed from February until June, thus, with increasing daylight levels that may have influenced the outcomes (i.e., expectedly lower impact of the light-emitting glasses at night with higher light exposure during daytime). However, time and group were included as separate factors in our analysis without showing any statistically significant effects on the results. However, it would be interesting to perform a future study during wintertime, when daylight levels are substantially shorter and, thus, the potential of light exposure larger.

A clear limitation of our study was the relatively low rate of intervention compliance (43% of all designated night shifts) The increased work pressure that the COVID-19 pandemic brought to the security service organization under study led to last-minute changes in duty (leave) schedules, amongst other things, which may have contributed to the low compliance. However, recent light exposure studies with high compliance rates (above 80%) also showed contradictory findings [15,17]. A low intervention compliance combined with a 11% lower actual sample size (n = 87) than originally calculated (n = 98), resulting in a post-hoc power of 0.62, may have dampened the potential effects. Irrespective of power, the observed effects are small, and clinical relevance is negligible, which can be an accurate reflection of the real effects when light glasses are used in a non-experimental setting.

In summary, exposure-related and study design-related factors are all intertwined when it comes to making an effective and feasible nocturnal light exposure protocol. In our field-based study setting, with approximately seven night shifts in a five week work schedule, and with an exposure duration of 30 min per night shift, light-emitting glasses did not have directly measurable effects on the need for recovery, overall fatigue or stress levels in a group of security guards. Future research is encouraged to optimize an adjusted nocturnal light exposure protocol, taking into account both short-term and longer-term outcome measures, in a larger target population. As suggested elsewhere [42], tailored protocols that take into account individual circadian timing may be needed in this respect.

## 4. Materials and Methods

### 4.1. Study Population

The study population consisted of actively serving civilian security guards from the Netherlands Armed Forces Surveillance and Security Organization, aged 18–67 years. Volunteers were recruited from seven patrol areas across the country. Individuals with a disease of the retina (e.g., macular degeneration) were excluded from the study, as were individuals who were not capable of fulfilling their regular duty tasks without wearing their own glasses for at least 30 min.

### 4.2. Design and Research Protocol

A randomized controlled cross-over trial was performed. The included study participants were randomly divided into two groups: one group (G1) used blue light-emitting glasses in a 5 week block schedule, which was followed by a standard 5 week block schedule without glasses. Conversely, the second group (G2) started with a standard five week block schedule without glasses, followed by a 5 week block schedule with the glasses.

The study was carried out between February and June 2021, using the organization’s regular work block schedule, which follows the Dutch standards on night work [14]. This schedule can be labelled as a rapidly rotating shift schedule [15], consisting of a 35 day (5 weeks) cycle that includes: 5 night shifts of 8 h, 2 night shifts of 12 h, 7 day shifts of 8 h, 2 day shifts of 12 h, 5 evening shifts of 8 h, and 14 days off. Each block schedule, thus, comprises 7 night shifts: one block of 3 consecutive nights shifts and two blocks of two consecutive night shifts. See Table 5 for the block schedule used in this study.

After the initial selection of the patrol areas, participants were informed in writing and orally about the purpose and duration of the study. Upon their decision to participate, an informed consent form was signed. Randomization was applied per patrol area to allocate participants to either G1 or G2. Measurements were taken at baseline in week (T_0_), after the first 5 week block schedule in week 5 (T_5_), and after the second 5 week block schedule in week 11 (T_11_).

### 4.3. Light Emitting Glasses Protocol

In this study, commercially available blue light glasses (Propeaq Premium Light Glasses Model 1.0B, Chrono Eyeware BV, Tilburg, The Netherlands) were used, containing integrated LEDs in the frame (λ = 461 ± 10 nm, spectral irradiance of 22.34 μW/cm^2^, melanopic EDI of 114.38 lx at the cornea of the eyes) [20]. All participants received extensive instructions on the use of the glasses and its underlying working mechanisms. Based on the participant’s personal work schedule and chronotype, tailored user instructions for wearing the light-emitting glasses during the 5 week intervention period were provided by a smartphone app developed by the light glass company.

Participants were instructed to wear the light-emitting glasses for 30 min during the first half of each night shifts in the intervention period, following the instructions provided by the manufacturer: light glasses need to be worn for 30 consecutive minutes, unless work activities were compromized. In the latter case, participants were instructed to use the glasses two times, for a total of 15 min within one hour. The app provided the time frame per night in which the light-emitting glasses should be used. In general, the light-emitting glasses were used between 11 pm and 1 am during the first night shift. For the second night shift, as well as for the weekend night shifts, the light-emitting glasses were used between 12 and 2 am. Participants had the freedom to choose when to wear the glasses within the 2 h time slot. Furthermore, the participants were advised not to use the light-emitting glasses at moments other than advised by the app. The study participants were allowed to use their usual coping strategies, such as caffeine intake. Naps were prohibited due to management policies.

In a position statement of 2019, the International Commission on Illumination (CIE) reported that the effects of blue light are harmless for healthy individuals, as no long-term side effects have been reported thus far [43]. The only contraindication for the use of light-emitting glasses are diseases that involve the retina of the eye (e.g., diabetes).

### 4.4. Measurements

The focus in the current study was on self-assessed functioning scores related to individual work-induced and general fatigue over a period of time, as they were considered more relevant outcome parameters to the security service context compared to momentary sleep-related improvements.

We chose the self-perceived need for recovery in the preceding 14 days as our primary outcome, as measured with the 11-item Need for Recovery Scale (NFRS). The NFRS is derived from the Dutch Questionnaire on the Experience and Evaluation of Work [44]. Higher scores indicate an increased need for recovery, which is used as an indication of work-related fatigue.

The secondary outcome measures included self-assessed general fatigue, motivation, concentration, and physical activity in the preceding 14 days, as measured with the Checklist Individual Strength (CIS) [45]. A higher overall score refers to an increased general fatigue, and decreased motivation, concentration, and physical activity levels, which represent the CIS subscales. The CIS is used to provide an indication of general fatigue and associated behavioural aspects.

Furthermore, indications of stress and (lack of) physiological recovery were measured using the parameter Stress Level from a commercially available fitness tracker (VivoSmart 4, Garmin, Olathe, KS, USA). This parameter is derived from measurements of heart rate and its derivative heart rate variability, respiratory rate and duration and intensity of body movements. Participants were instructed to wear the fitness tracker for 5 consecutive days after each measurement at week T_0_, T_5_, and T_11_; averaged 5 day values were used.

After the completion of the post-intervention measurement at T_5_ for G1 and T_11_ for G2, participants were asked to complete additional questionnaire items on intervention compliance; self-perceived effectiveness; credibility of the intervention; and ease of use of the light-emitting glasses. At baseline, chronotype was measured as a potential covariate, using one item of the reduced Morningness–Eveningness Questionnaire (rMEQ-5), as suggested by Loureiro & Garcia-Marques [46]. Moreover, age, years in service, and self-reported use of sleep-stimulating aids and medications in the previous month were assessed at baseline.

### 4.5. Sample Size Calculation

The sample size was based on the NFRS score changes. As a reference population for the current study population, ‘Subpopulation B’, from the Maastricht Cohort Study on Fatigue at Work, was used. This subpopulation consisted of approximately 2000 shift workers with different work schedules and a low to medium educational level [47], which is comparable to the average education level of the population under study. The mean baseline of the NFRS overall score of the reference population was 38.4 (standard deviation 27.2). To detect a relevant 20% decrease in NFRS scores, with a power of 80%, and a significance level of 0.05, 98 participants were needed in our study.

### 4.6. Data Analysis

The data were analysed using IBM SPSS Statistics Version 27. Descriptive statistics were used to describe basic features of the study population. A mixed model with 3 repeated measures was used to test group-related and time-related intervention effects for NFRS and CIS. Chronotype was included in the model as a covariate. Akaike information criterion (AIC) was used for assessing goodness of fit of the mixed-effects model with random intercept.

Stress level scores were processed and analysed after uploading the data from the trackers onto an online data platform (FitRockr Analytics Platform, Berlin, Germany). Pre-postintervention stress values were analysed with Paired-Samples T Tests.

### 4.7. Handling of (Missing) Data

Pseudonymized data were collected and merged using participant numbers, and were analysed following the intention-to-treat principle. Missing NFRS questionnaire data were handled according to the available questionnaire-specific instructions, which stated that the sum score should be calculated by dividing the available data points by the number of correctly answered questionnaire items [48]. For missing data in the CIS subscales, the mean value of all other available subscale data were imputed. For missing tracker data, a listwise deletion was applied in case of MCAR.

## Figures and Tables

**Table 1:** Baseline characteristics of the study participants. Independent T-tests were used to assess differences between means of both groups at baseline (G1–G2).

	Total Group	G1	G2	*p* Value G1–G2
Participants (N)	87	45	42	
Sex (%):				
Male	82 (94%)	42 (93%)	40 (95%)	
Female	5 (6%)	3 (7%)	2 (5%)	
Age groups (N, %)				
21–30 years	10 (12%)	3 (7%)	7 (17%)	
31–40	40 (46%)	23 (51%)	17 (40%)	
41–50	27 (31%)	13 (29%)	14 (33%)	
51–60 Mean (SD)	10 (11%) 39.6 (8.1)	6 (13%) 40.0 (8.0)	4 (10%) 39.2 (8.3)	0.705
Number of years in service:				
1–9 years (N, %)	20 (23%)	8 (18%)	12 (29%)	
10–19	44 (51%)	25 (56%)	19 (45%)	
20–29	16 (18%)	7 (16%)	9 (21%)	
30–38 Mean (SD)	7 (8%) 15.3 (8.7)	5 (11%) 16.3 (8.8)	2 (5%) 14.2 (8.6)	0.986
Use of sleep medicine (N, %)				
Prescribed by GP	8 (9%)	4 (9%)	4 (10%)	
Other (e.g., melatonin)	10 (12%)	6 (13%)	4 (10%)	
Chronotype (N, %)				
Clearly morning type	13 (15%)	5 (11%)	8 (19%)	
More morning than evening type	20 (23%)	13 (29%)	7 (17%)	
More evening than morning type	23 (26%)	12 (27%)	11 (26%)	
Clearly evening type	31 (36%)	15 (33%)	16 (38%)	
Outcomes (mean, SD):				
NFRS	27.6 (23.7)	22.2 (19.5)	33.5 (26.3)	0.044
CIS	59.2 (20.9)	57.5 (19.1)	60.6 (22.9)	0.129

Note. N = numbers, SD = standard deviation, GP = general practitioner, NFRS = Need for Recovery, CIS = Checklist Individual Strength.

**Table 2:** Mean outcome scores of G1 (N = 45) at baseline (T_0_), after five weeks of wearing light-emitting glasses during night shifts (T_5_), followed by five weeks of not wearing glasses (T_11_).

G1	NFRS		CIS		Stress Level	
	Mean	SD	Mean	SD	Mean	SD
T_0_	22.2	19.5	57.5	19.1	-	-
T_5_	21.2	22.1	57.4	19.0	30.5	8.9
T_11_	21.0	24.2	56.5	20.7	34.3	10.5

**Table 3:** Mean outcome scores of G2 (N = 42) at baseline (T_0_), after five weeks of not wearing light-emitting glasses during night shifts (T_5_), followed by five weeks of wearing glasses (T_11_). One participant dropped out before T_11_.

G2	NFRS		CIS		Stress Level	
	Mean	SD	Mean	SD	Mean	SD
T_0_	33.5	26.3	60.6	22.9	-	-
T_5_	32.2	28.2	56.4	24.8	32.9	15.8
T_11_	30.4	31.2	55.9	24.7	33.9	9.9

**Table 4:** Results from a Mixed model analyses with three repeated measures for NFRS and CIS.

	NFRS		CIS	
	F	*p* Value	F	*p* Value
Time	0.662	0.517	3.058	0.050
Intervention sequence	4.409	0.039	0.005	0.943
Time * Intervention sequence	0.246	0.783	2.223	0.111

**Table 5:** Regular work schedule used by participating security guards; night shifts are shaded.

Day Week	Monday	Tuesday	Wednesday	Thursday	Friday	Saturday	Sunday
1	D	D	E	E	E	−	−
2	−	−	D	D	N	WN	WN
3	−	−	PST	PST	D		
4	N	N	−	−	−	WD	WD
5	E	E	N	N	−	−	−

**D**, Day shift from 06:00–14:15; **E**, Evening shift from 14:00–22:15; **N**, Night shift from 22:00–06:15; **WD**, Weekend Day shift from 06:00–18:15; **WN**, Weekend Night shift from 18:00–06:15; **PST**, Professional Skills Training from 06:00–14:15; **−**, Planned leave.

## Data Availability

The data presented in this study are available on request from the corresponding author. The data are not publicly available due to organizational policy.
